# PAS Domain Protein Pas3 Interacts with the Chromatin Modifier Bre1 in Regulating Cryptococcal Morphogenesis

**DOI:** 10.1128/mBio.02135-18

**Published:** 2018-11-13

**Authors:** Youbao Zhao, Srijana Upadhyay, Xiaorong Lin

**Affiliations:** a Department of Microbiology, University of Georgia, Athens, Georgia, USA; b Department of Biology, Texas A&M University, College Station, Texas, USA; Duke University Medical Center

**Keywords:** morphogenesis, PAS domain, histone modification, epigenetic, mating, subcellular localization, transcriptional regulation

## Abstract

Switching between different morphotypes is an adaptive cellular response in many microbes. In Cryptococcus neoformans, the yeast-to-hypha transition confers resistance to microbial predation in the soil and is an integral part of its life cycle. Morphogenesis is also known to be associated with virulence, with the filamentous form being immune-stimulatory and protective in mammalian models of cryptococcosis. Previous studies identified the transcription factor Znf2 as a master regulator of cryptococcal filamentation. However, the upstream regulators of Znf2 remain largely unknown. PAS domain proteins have long been recognized as transducers of diverse environmental signals. Here, we identified a PAS domain protein Pas3 as an upstream regulator of Znf2. Surprisingly, this small Pas3 protein lacks a nuclear localization signal but is enriched in the nucleus where it regulates the transcript level of *ZNF2* and its prominent downstream targets. We discovered that the PAS domain is essential for Pas3’s nuclear enrichment and function. Intriguingly, Pas3 interacts with Bre1, which is required for *Cryptococcus* histone H2B monoubiquitination (H2Bub1) and H3 lysine 4 dimethylation (H3K4me2), two histone modifications known to be associated with active gene transcription. Indeed, Bre1 functions together with Pas3 in regulating cryptococcal filamentation based on loss-of-function, epistasis, and transcriptome analysis. These findings provide the first evidence of a signaling regulator acting with a chromatin modifier to control cryptococcal filamentation.

## Abstract (precis)

For the ubiquitous environmental pathogen Cryptococcus neoformans, the morphological transition from yeast to filament confers resistance to natural predators like soil amoeba and is an integral differentiation event to produce infectious spores. Interestingly, filamentation is immuno-stimulatory and attenuates cryptococcal virulence in a mammalian host. Consistently, the morphogenesis transcription factor Znf2 profoundly shapes cryptococcal interaction with various hosts. Identifying the signaling pathways activating filamentation is thus, conductive to a better understanding of cryptococcal biology. In this study, we identified a PAS domain protein Pas3 that functions upstream of Znf2 in regulating cryptococcal filamentation. Interestingly, Pas3 interacts with the chromatin modifier Bre1 in the nucleus to regulate the transcript level of Znf2 and its prominent downstream targets. This is the first example of a PAS domain signaling regulator interacting with a chromatin modifier to control filamentation through their impact on cryptococcal transcriptome.

## INTRODUCTION

Cryptococcus neoformans is the major cause of life-threatening fungal meningoencephalitis (1), responsible for ∼180,000 deaths each year (2). This environmental microbe infects various hosts, ranging from single-cell amoeba to insects, plants, and mammals. This fungus is primarily found in soil contaminated with pigeon droppings or decaying vegetation (3). One of the prominent adaptive responses in this ubiquitous microbe is the morphological transition (4). C. neoformans switches from the yeast form to the hyphal form in response to nutrient limitation, dehydration, predation, and plant hormones (5–7). Filaments are resistant to predation by cryptococcal natural predators such as soil amoeba (8). Furthermore, aerial hyphae could subsequently differentiate into fruiting bodies, which produce stress-tolerant propagules for dispersal and infection (9, 10).

Switching morphotype from yeast to hypha is a tightly regulated cellular response, with the upstream signaling pathways integrating the external signals or physiological changes to trigger morphogenesis. The transcription factor Znf2 is a downstream regulator essential for the initiation and maintenance of filamentous growth, be it dikaryotic hyphal growth during bisexual mating or self-filamentation during unisexual development (11, 12). However, the signaling pathways upstream of Znf2 remain largely unknown, except the highly conserved pheromone sensing and response pathway.

PAS proteins have long been recognized as signaling regulators that monitor physiological and environmental changes (13, 14). PAS is an acronym representing the three proteins (PER-ARNT-SIM) in which the PAS repeat sequences were first identified (13). PAS proteins have been identified in the unique two-component system (TCS)-like upstream sensor modules of the high-osmolarity glycerol response (HOG) pathway (15), in the white collar complex (WCC) required for blue light sensing (16), and in phytochrome responsible for red light sensing (17, 18). In Cryptococcus, the WCC composed of Bwc1 and Bwc2 suppresses mating in response to blue light, whereas the Phy1 phytochrome plays no apparent role in mating or filamentation (19).

In this study, we examined all nine PAS proteins encoded in the C. neoformans genome and discovered that Pas3 functions upstream of Znf2 in controlling morphogenesis. Interestingly, Pas3 is enriched in the nucleus and the PAS domain is critical for its recruitment to the nucleus. Pas3 interacts with the E3 ubiquitin ligase Bre1, which affects histone modifications that promote active transcription. Pas3 and Bre1 cooperate to regulate filamentation-associated genes through their influence on the cryptococcal transcriptome. This is the first example of a signaling regulator interacting with a chromatin modifier to control cryptococcal filamentation.

## RESULTS

### Phylogenic analysis of cryptococcal PAS domain-containing proteins.

The PAS domain typically shows conserved functions in sensing and transducing environmental signals in various organisms. We decided to identify and analyze all of the PAS proteins present in diverse fungal species (Table 1). All of the fungal species searched contain multiple PAS proteins. Three PAS proteins were present in Saccharomyces cerevisiae and Candida albicans, while more were found in *Sordariomycetes* (13 in Fusarium graminearum and 14 in both Magnaporthe oryzae and Neurospora crassa). An intermediate numbers of PAS proteins were found in the selected *Basidiomycetes* species (nine in C. neoformans, seven in Ustilago maydis, and six in Coprinopsis cinerea).

**TABLE 1:** PAS domain proteins in fungi

Phylum and species	No. of PAS proteins	Gene ID
*Ascomycota*		
Aspergillus fumigatus	7	Afu4g12690, Afu6g10240, Afu3g05780, Afu2g15010, Afu3g12530, Afu4g02900, Afu6g09260
Candida albicans	3	C3_06620W_A, CR_03050C_A, C7_00740W_A
Saccharomyces cerevisiae	3	YAL017W, YBR239C, YPL133C
Fusarium graminearum	13	FGSG_08456, FGSG_00710, FGSG_00737, FGSG_00856, FGSG_01312, FGSG_01943, FGSG_17004, FGSG_08781, FGSG_02972, FGSG_17624, FGSG_17634, FGSG_05990, FGSG_07941
Magnaporthe oryzae	14	MGG_11882, MGG_07517, MGG_01041, MGG_00345, MGG_00295, MGG_02665, MGG_06026, MGG_08735, MGG_03538, MGG_04521, MGG_01227, MGG_01342, MGG_13891, MGG_12377
Neurospora crassa	14	NCU03967, NCU03938, NCU07221, NCU02356, NCU04834, NCU00902, NCU07268, NCU06390, NCU00939, NCU03164, NCU02057, NCU01833, NCU07378, NCU04834
Schizosaccharomyces pombe	6	SPAC1834.08, SPCC74.06, SPAPB18E9.02c, SPAC1805.01c, SPAC27E2.09, SPCC1450.11c

*Basidiomycota*		
Cryptococcus neoformans	9	CNAG_01988, CNAG_02435, CNAG_03024, CNAG_03355, CNAG_04271, CNAG_04588, CNAG_05181, CNAG_05590, CNAG_06200
Ustilago maydis	7	UMAG_10450, UMAG_06278, UMAG_02052, UMAG_02664, UMAG_03180, UMAG_11957, UMAG_05732
Coprinopsis cinerea	6	CC1G_03210, CC1G_06391, CC1G_14606, CC1G_03335, CC1G_02967, CC1G_08609

**FIG 1:**
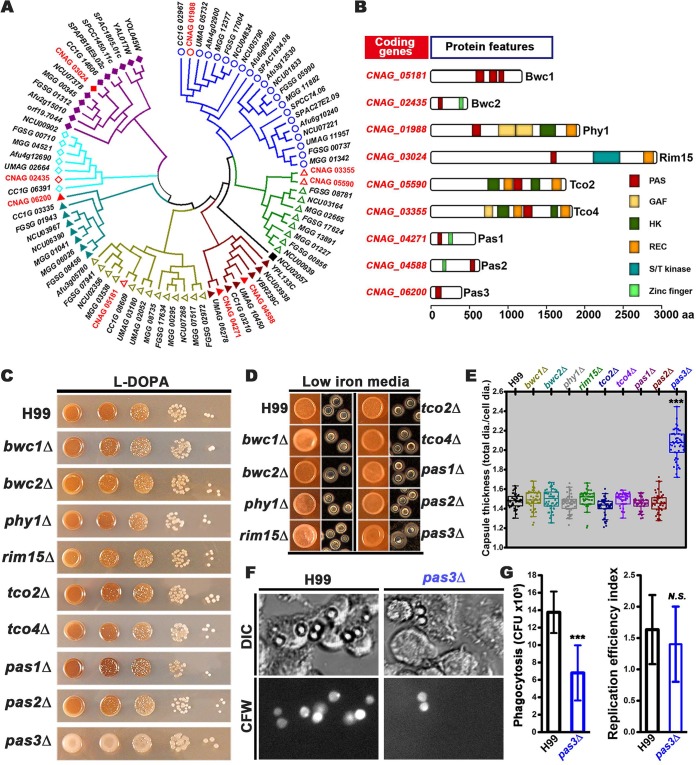
PAS proteins are phylogenetically conserved in fungi, and Pas3 regulates cryptococcal virulence traits. (A) A phylogenetic tree of PAS proteins in selected fungi generated based on their predicted protein sequences. C. neoformans PAS proteins are marked in red. (B) A diagram depicting the domain structures of PAS proteins in C. neoformans. aa, amino acids. (C) WT H99 and the *PAS* gene deletion mutants were cultured on l-DOPA medium for melanization. (D) Image of the colony (left) and individual cells (right) with Indian ink of H99 and *PAS* gene deletion mutants grown on the capsule-inducing low-iron medium (LIM). (E) Quantification of capsule thickness from all strains by the ratio of total diameter versus cell diameter. A total of 50 cells of each strain were measured. (F) Phase and fluorescent images of phagocytosis of H99 and *pas3*Δ cells. Murine J774A.1 cells were infected by C. neoformans wild-type strain H99 and *pas3*Δ mutant for two hours, fixed, and then stained with calcofluor white (CFW). (G) Statistical analysis on the percentage of phagocytosed C. neoformans cells and the replication efficiency of H99 and *pas3*Δ cells within macrophage cells. ***, 0.001; *N.S.*, not significant.

We further analyzed the phylogenetic relationship between the PAS proteins on the basis of whole-protein sequences. The PAS proteins from the selected fungal species were clustered into seven clades, with nine cryptococcal PAS proteins distributed within these seven clades (Fig. 1A). The PAS proteins clustered in the same clade, irrespective of the evolutionary distance between the host organisms, share similar domain architectures based on the known or predicted protein features (Fig. 1B).

### PAS3 affects cryptococcal virulence traits.

To examine the functions of cryptococcal PAS proteins, we generated *pas3*Δ mutants and obtained the remaining eight *PAS* gene deletion mutants in the H99 background from the partial-genome deletion set generated by Hiten Madhani’s laboratory. We first tested these strains for their susceptibility to various stresses. The *PAS* gene deletion mutants exhibited susceptibility similar to that exhibited by the wild-type (WT) strain with respect to high temperature (37°C), osmotic stress (1.5 M NaCl), and Congo red-induced cell wall stress (see Fig. S1A in the supplemental material). The *bwc1*Δ and *bwc2*Δ mutants were markedly more sensitive to UV irradiation, and the *rim15*Δ and the *tco4*Δ mutants were more sensitive to fludioxonil (100 μg/ml), consistent with previous reports (15, 19, 20). All of the other *PAS* gene deletion mutants showed sensitivity comparable to that shown by the WT strain (Fig. S1A).

10.1128/mBio.02135-18.1 FIG S1 The *pas3*Δ mutant was not susceptible to tested stresses and showed lung fungal burden comparable to that seen with the corresponding wild-type controls. (A) To test the ability of the strains to grow at high temperatures and their sensitivity to UV radiation, serially diluted cells (starting from OD_600_ = 3) were spotted onto YNB agar medium and grown at 37°C with or without exposure to 300 J/m^2^ of UV (3s). To test the tolerance of fungal cells with respect to osmotic and other stressors, cells were grown at 22°C for 2 days on YPD medium with or without supplementation with H_2_O_2_ (21 mM), NaCl (1.5 M), KCl (1 M), fludioxonil (100 μg/ml), or Congo red (200 μg/ml). (B) Mice (5 per group) were intranasally infected with 1 × 10^4^ yeast cells for serotype A strains and 1 × 10^6^ yeast cells for serotype D strains. The mice were sacrificed after 12 days postinfection (DPI 12) by serotype A strains and 17 days postinoculation (DPI 17) by serotype D strains. The lungs from sacrificed mice were dissected and homogenized in phosphate-buffered saline, serially diluted, and plated on YNB plates for counting CFU. Download FIG S1, PDF file, 0.3 MB. Copyright © 2018 Zhao et al. 2018 Zhao et al. This content is distributed under the terms of the Creative Commons Attribution 4.0 International license.

Melanin (21), capsule (22), and the ability to grow at the human body temperature (23) are three well-established virulence traits critical for cryptococcal adaptation to a mammalian host. Melanin is a brown-black pigment that protects the fungus from UV irradiation (24) in the environment and from toxic free radicals produced by the host defense systems (25–27). The polysaccharide capsule surrounding cryptococcal cells protects the fungus from dehydration in nature and mediates its interactions with the host (28–30). The deletion of *PAS3*, but not any other *PAS* genes, significantly reduced melanization (Fig. 1C). Likewise, the deletion of *PAS3*, but not any other *PAS* genes, significantly increased the capsule size (Fig. 1D and E). These results indicate that Pas3 promotes melanization but suppresses capsule enlargement.

The cryptococcal capsule is known to suppress phagocytosis of C. neoformans by macrophages (28, 31). When the murine J774A.1 macrophages were infected with wild-type H99 and the *pas3*Δ mutant, we found reduced phagocytosis but no significant difference in the level of intracellular replication of mutant *pas3*Δ (Fig. 1F and G), possibly due to its increased capsule size. To determine if Pas3 affects cryptococcal pathogenicity, we infected mice with *pas3*Δ mutants generated in both serotype A H99 and serotype D XL280 backgrounds. We examined the fungal burden in the lungs at day 12 postinfection by serotype A strains and at day 17 postinfection by serotype D strains. We found comparable levels of fungal load between the *pas3*Δ mutants and the corresponding wild-type controls (Fig. S1B). The lack of impact on the lung fungal burden by the deletion of *PAS3* could have been due to its opposing effects on melanization and capsule size.

### PAS3 regulates cryptococcal cell fusion and filamentation.

**FIG 2:**
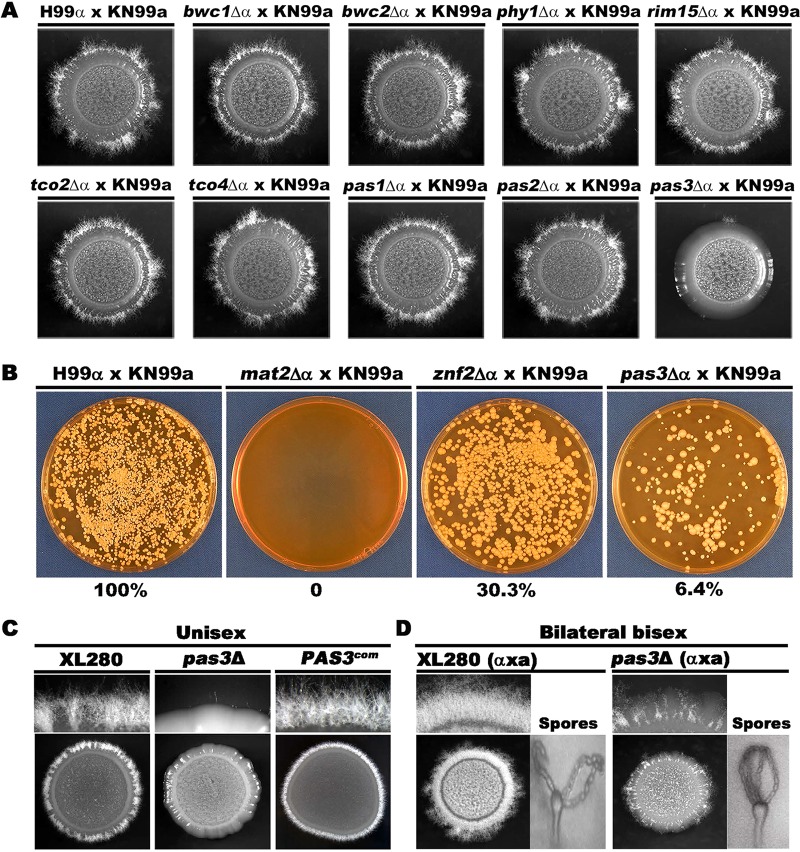
Pas3 regulates cryptococcal cell fusion and filamentation. (A) Colony morphology of the cross between α isolates (WT H99 and PAS mutants) and WT KN99**a** strain on V8 medium at 22°C in dark for 14 days. (B) Cell fusion products of the indicated crosses at 48 h postcoculturing on V8. (C) WT XL280 and the *pas3*Δ mutant (OD_600_ = 3) were spotted on V8 medium to test self-filamentation during unisexual development. (D) The bilateral mating of the wild-type partners (XL280α × XL280**a**) and the *pas3*Δ mutants (*pas3*Δα × *pas3*Δ**a**) on V8 medium at 22°C in dark for 10 days.

To determine if any of the PAS proteins play important roles in cryptococcal morphogenesis, we set up unilateral bisexual crosses (mutant × wild type) where the *PAS* mutants of mating type α were cocultured with equal numbers of the wild-type partner of mating type **a** on V8 juice agar medium. As expected, the cross between the wild-type α and wild-type **a** mating partners generated robust mating hyphae under this condition. Robust filamentation was also observed from crosses between the wild-type strains and the *PAS* gene deletion mutants, except for mutant *pas3*Δ. The cross between mutant *pas3*Δ and strain KN99**a** showed sporadic filamentation only after prolonged incubation (Fig. 2A).

The defect of mutant *pas3*Δ in hyphal production during unilateral bisexual mating could have been caused by impaired cell fusion or by impaired filamentation *per se* or both. During bisexual α-**a** mating, mating hyphae are initiated from the fused α/**a** zygotes (32, 33). A defect in cell fusion consequently causes defective production of mating hyphae. If cell fusion occurs, a defect in filamentation *per se* in one of the mating partner usually results in a reduction in filamentation due to a gene dosage effect.

To test these hypotheses, we examined cell fusion efficiency of the unilateral cross between the *pas3*Δ α mutant and a wild-type **a** partner at 48 h postcoculturing on V8 juice agar medium. The crosses of strains α × **a**, *mat2*Δ α × **a**, and *znf2*Δ α × **a** were included as controls. The *mat2*Δ mutant cannot undergo cell fusion due to the disruption of the pheromone sensing and response pathway (11). The *znf2*Δ mutant cannot filament even after successful cell fusion during bilateral mating because of the defect in morphogenesis (11). As expected, deletion of *MAT2* abolished cell fusion (0%), while deletion of *ZNF2* modestly (30.3%) reduced cell fusion in the unilateral cross (Fig. 2B). Deletion of *PAS3* in the mating type α background caused a drastic reduction (6.4% of the wild-type level) in cell fusion in crosses performed with a WT mating type **a** strain. A comparable reduction in cell fusion frequency was also observed when *PAS3* was deleted in the mating type **a** partner (WT α × mutant *pas3*Δ **a** in Fig. S2A). Taking the data together, these results indicate that Pas3 regulates cell fusion in a mating type-independent manner in C. neoformans.

10.1128/mBio.02135-18.2 FIG S2 Pas3 regulates cell fusion in a mating type-independent manner and is localized to the nucleus in both the WT and the *znf2*Δ mutants. (A) Reductions in cell fusion frequency caused by the deletion of *PAS3* are mating type independent. Reciprocal cell fusion assays were done between mutant *pas3*Δ α or mutant *pas3* Δ ****a**** and the corresponding wild-type strain, H99 α or KN99 a. (B) Strains with mCherry-tagged Pas3 in the wild-type and *znf2*Δ backgrounds were cultured overnight in liquid YPD at 30°C with shaking, and fluorescent signals were observed under a Zeiss Imager M2 microscope. Download FIG S2, PDF file, 0.1 MB. Copyright © 2018 Zhao et al. 2018 Zhao et al. This content is distributed under the terms of the Creative Commons Attribution 4.0 International license.

To examine if Pas3 also regulates filamentation *per se*, we deleted *PAS3* in serotype D reference strain XL280. XL280 can undergo self-filamentation without the need of cell fusion (34–36). Interestingly, self-filamentation was almost abolished in the *pas3*Δ mutant (Fig. 2C). Introduction of the wild-type copy of *PAS3* driven by its native promoter into mutant *pas3*Δ restored filamentation (Fig. 2C). These results indicate that Pas3 regulates cryptococcal filamentation as well as cell fusion.

As filamentation precedes sporulation during sexual reproduction in C. neoformans and Pas3 regulates cell fusion and filamentation, we decided to examine if Pas3 also regulates sporulation. We generated the *pas3*Δ mutant in the congenic XL280**a** background (37) and crossed it with the *pas3*Δ mutant in the XL280α background. Hypha production was severely impaired during mutant *pas3*Δ α × *pas3*Δ **a** bilateral mating (Fig. 2D), consistent with the role of Pas3 in regulating cell fusion and filamentation. Interestingly, fruiting body differentiation and sporulation occurred normally in the spotty filamentous areas in the bilateral cross (mutant *pas3*Δ α × *pas3*Δ **a**) (Fig. 2D). These results indicate that Pas3 is not critical for fruiting body differentiation or sporulation.

### PAS3 functions upstream of Znf2 in regulating filamentation.

**FIG 3:**
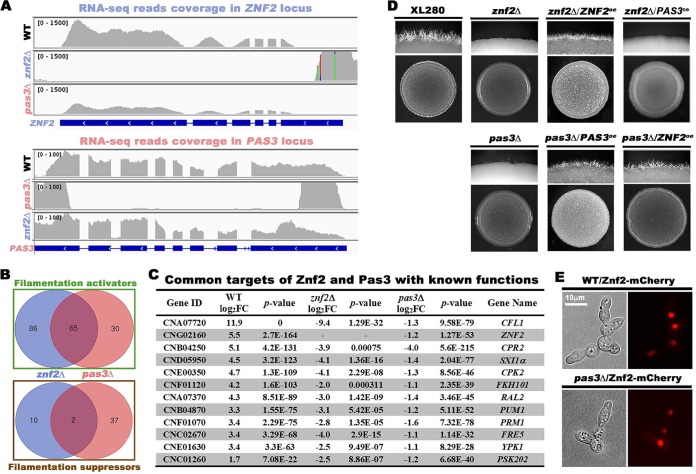
Pas3 functions upstream of Znf2, and it regulates Znf2 and Znf2’s prominent downstream targets at the transcript level. (A) RNA sequencing read coverage over the *ZNF2* locus and the *PAS3* locus in the corresponding mutant *znf2*Δ and *pas3*Δ backgrounds. (B) Venn diagrams of the filamentation-associated differentially expressed (DE) genes in the transcriptome of mutants *znf2*Δ and *pas3*Δ. (C) Gene function and fold change of filamentation-associated genes with known functions that are commonly regulated by Znf2 and Pas3. (D) *PAS3* and *ZNF2* were reciprocally overexpressed in the *znf2*Δ and the *pas3*Δ mutants. The overexpression strains and control strains (OD_600_ = 3) were spotted onto V8 medium to test self-filamentation. (E) Localization of mCherry-tagged Znf2 in WT and the *pas3*Δ mutant.

Znf2 is the key transcription factor controlling cryptococcal filamentation (11). To probe the relationship between Pas3 and Znf2, we performed comparative transcriptome analysis among WT, *znf2*Δ, and *pas3*Δ strains using RNAs from these strains cultured on V8 medium or filamentation-suppressing yeast extract-peptone-dextrose (YPD) medium. No reads were mapped to the *ZNF2* locus in mutant *znf2*Δ or the *PAS3* locus in mutant *pas3*Δ (Fig. 3A), verifying the strains used. Interestingly, the read coverage of *ZNF2* was significantly reduced in mutant *pas3*Δ, while the read coverage of *PAS3* in mutant *znf2*Δ was comparable to that seen with the wild-type control (Fig. 3A). These results indicate that *PAS3* may function upstream of *ZNF2* in regulating its transcript level.

To further investigate the genetic relationship between Znf2 and Pas3 in regulating cryptococcal filamentation, we planned to identify the filamentation-associated genes by comparing the transcriptomes of the wild-type strain cultured on YPD and V8 media and then comparing the filamentation-associated regulons that are affected by Znf2 and Pas3 on V8 medium. First, we analyzed the WT strain cultured on V8 medium in comparison to WT on YPD medium. A total of 2,099 of about 7,000 *Cryptococcus* protein-coding genes exhibited statistically significant differential expression results (|log2 fold change [log2FC]| > 1; adjusted *P* value < 0.05), with 1,075 genes upregulated and 1,024 genes downregulated on V8 medium. Changes at this magnitude reflect dramatic remodeling of the cryptococcal transcriptome under these conditions (see Table S2 in the supplemental material). We postulate that the activators of filamentation may be enriched in the pool of upregulated genes on V8 medium, while the suppressors of filamentation may be enriched in the pool of downregulated genes.

Next, we decided to identify the regulator-dependent targets under the filamentation-inducing condition. We analyzed the transcriptomes of mutants *znf2*Δ and *pas3*Δ cultured on V8 medium against that of the WT strain grown on V8 medium. Few genes were affected by the deletion of *ZNF2* when C. neoformans was cultured on YPD medium where both the WT and *znf2*Δ strains were growing in the yeast form. This is consistent with Znf2 being a filamentation-specific regulator. By contrast, the expression levels of 268 genes were significantly changed in mutant *znf2*Δ compared to the WT on V8 medium (|log2FC| > 1, adjusted *P* value < 0.05), with 72 genes significantly upregulated and 196 genes downregulated. The two pairwise analyses (WT V8 versus WT YPD and mutant *znf2*Δ V8 versus WT V8) yielded two pools of differentially expressed genes. We postulate that Znf2-dependent activators of filamentation would be enriched in the upregulated pool from the pairwise analysis of WT V8 versus WT YPD and in the downregulated pool from the pairwise analysis of mutant *znf2*Δ V8 versus WT V8. Conversely, Znf2-dependent suppressors would be enriched in the opposite pools. Accordingly, we identified 151 potential activators and 12 potential suppressors of filamentation regulated by Znf2 through this intersection analysis (Table S3). By the same approach, we analyzed the transcriptome data from the *pas3*Δ mutant. The expression of 317 genes was significantly changed in mutant *pas3*Δ compared to the WT on V8 medium, (|log2FC| > 1, adjusted *P* value < 0.05), with 189 genes significantly upregulated and 127 genes downregulated. Together with the transcriptomes of WT V8 versus WT YPD, we identified 95 potential activators and 39 potential suppressors of filamentation regulated by Pas3 (Table S4).

Intersection analysis of the filamentation activators regulated by Znf2 (151 genes) and Pas3 (95 genes) indicated that 65 genes were shared (Fig. 3B). Among the filamentation activators affected by the deletion of *PAS3* were *ZNF2* and known targets of Znf2 such as *CFL1* and *PUM1* (12, 38, 39) (Fig. 3C). These results again support the idea that Pas3 functions upstream of Znf2, regulating the transcript level of *ZNF2* and Znf2’s prominent downstream targets. In contrast to the activators, only two potential filamentation suppressors were identified as targets shared by Znf2 and Pas3 (Fig. 3B), suggesting that Znf2 and Pas3 promote cryptococcal filamentation mainly through activating the filamentation machinery.

To examine the genetic relationship between *PAS3* and *ZNF2*, we performed reciprocal overexpression analyses of *PAS3* and *ZNF2* in the *znf2*Δ and *pas3*Δ mutants. Overexpression of *PAS3* failed to confer filamentation to the *znf2*Δ mutant, while overexpression of *ZNF2* restored filamentation in the *pas3*Δ mutant (Fig. 3D). Collectively, the results from the transcriptome and epistasis analyses indicate that Pas3 functions upstream of Znf2 in regulating filamentation.

### PAS3 localizes to the nucleus and does not regulate Znf2 at the protein level.

**FIG 4:**
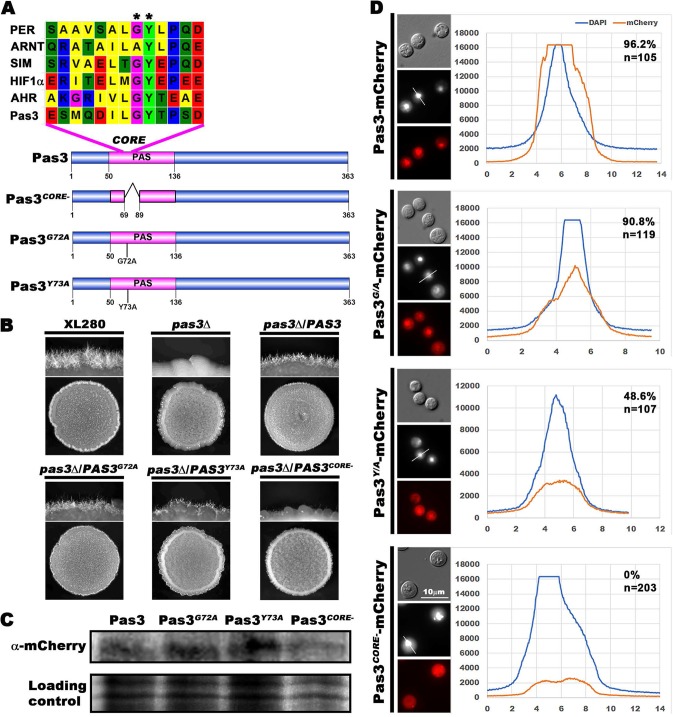
The PAS domain is important for the nuclear enrichment of Pas3 and its full function in regulating filamentation. (A) Diagram of Pas3 alleles with mutation or deletion in the core of PAS domain of Pas3. ClustalW multiple alignment of the PAS domain amino acid sequences in Per (AAH72458.1), Arnt (NP_001284648.1), Sim (AAB62395.1), HIF1α (NP_001521.1), Ahr (NP_001612.1), and Pas3 was performed. G72 and Y73 residues in Pas3 are indicated with stars. (B) Western blotting of mCherry-tagged Pas3 alleles probed with an anti-mCherry antibody. (C) Self-filamentation assay of WT, *pas3*Δ, *pas3*Δ/*PAS3*, *pas3*Δ/*PAS3^G72A^*, *pas3*Δ/*PAS3^Y73A^*, and *pas3*Δ/*PAS3^CORE^*^−^ strains. (D) Fluorescence of mCherry-tagged Pas3 alleles (images at bottom) and the quantitative analysis of their subcellular localization in the population. DAPI staining was used to visualize the nucleus (black-and-white images in the middle). The relative fluorescence intensities determined for DAPI and mCherry were plotted along the indicated lines. The percentages of cells with nucleus-enriched mCherry signal and the number of cells measured are indicated on the top right corner.

Given that overexpression of *ZNF2* restored filamentation in mutant *pas3*Δ (Fig. 3D), Pas3 is unlikely to regulate Znf2 posttranscriptionally. To test this hypothesis, we decided to artificially express *ZNF2* independently of *PAS3*. To this end, we transformed the inducible promoter-driven and mCherry-tagged *ZNF2* gene into the *pas3*Δ mutant. As we have shown previously (40), Znf2 is localized to the nucleus (Fig. 3E). We found that the nuclear localization of Znf2, once expressed, was unchanged in the absence of *PAS3* (Fig. 3E). This suggests that Pas3 does not regulate the subcellular localization of the Znf2 protein. It is likely that Pas3 regulates *ZNF2* primarily at the transcript level, implying that Pas3 may function in the nucleus. There is no nuclear localization signal (NLS) or DNA binding domain predicted in Pas3 (Fig. 4A; see also Fig. 1B), and we decided to generate mCherry-tagged Pas3 to examine its subcellular localization. Pas3-mCherry restored filamentation when expressed in the *pas3*Δ mutant (Fig. 4B), indicating its functionality. Pas3 was observed in the cytosol, but the fluorescence signals from 96.2% of cells were enriched in the nucleus (Fig. 4D). Localization of Pas3 in the *znf2*Δ mutant was indistinguishable from that in the wild type (Fig. S2B), indicating that Znf2 does not affect Pas3 subcellular distribution. This result is consistent with Pas3 functioning upstream of Znf2.

### The PAS domain is important for the function and subcellular localization of PAS3.

The PAS domain is the only recognizable domain in Pas3 (Fig. 4A). The core sequences of PAS domains in representative PAS proteins from different organisms carry the conserved glycine 72 (G72) and tyrosine 73 (Y73) residues (Fig. 4A). Mutations in these residues in the *Drosophila* Per protein cause dysfunction (41). To examine if the PAS domain is critical for its function and subcellular localization, we mutated conserved residue G72 or Y73 into alanine (A) and introduced the mCherry-tagged Pas3^G72A^ allele or Pas3^Y73A^ allele into mutant *pas3*Δ. Western blotting results showed that the two alleles were expressed at levels similar to those seen with the wild-type allele of Pas3 (Fig. 4C). In contrast to the wild-type allele of Pas3, Pas3^G72A^ and Pas3^Y73A^ restored filamentation in the *pas3*Δ mutant only partially (Fig. 4B), suggesting that these two residues are important for the full function of Pas3 in regulating filamentation. The majority (90.8%) of cells carrying Pas3^G72A^ retained nucleus-enriched signals, similarly to the wild-type Pas3 (96.2%) (Fig. 4D). By contrast, approximately half (48.6%) of the population showed nucleus-enriched localization of Pas3^Y73A^, and the fluorescent intensity was 3-fold to 4-fold lower than that of the wild type (Fig. 4D). These results indicate that the PAS domain is required for proper subcellular localization of Pas3. To further confirm the role of PAS domain in Pas3, we performed an in-frame deletion of the core sequence of PAS domain and introduced mCherry-tagged Pas3*^CORE^*^−^ into mutant *pas3*Δ. Western blotting results showed that Pas3*^CORE^*^−^ was expressed at a level similar to the levels seen with the wild-type allele and the other two mutated alleles of Pas3 in the *pas3*Δ background (Fig. 4C). Interestingly, Pas3*^CORE^*^−^ failed to restore the filamentation defect in mutant *pas3*Δ (Fig. 4B), and the mCherry-tagged Pas3*^CORE^*^−^ was found to be mostly diffused in the cytosol rather than being enriched in the nucleus (Fig. 4D). Taking the data together, the PAS domain is important for both localization and full function of Pas3 in *Cryptococcus*.

### Pas3 interacts with Bre1 to regulate cryptococcal filamentation.

**FIG 5:**
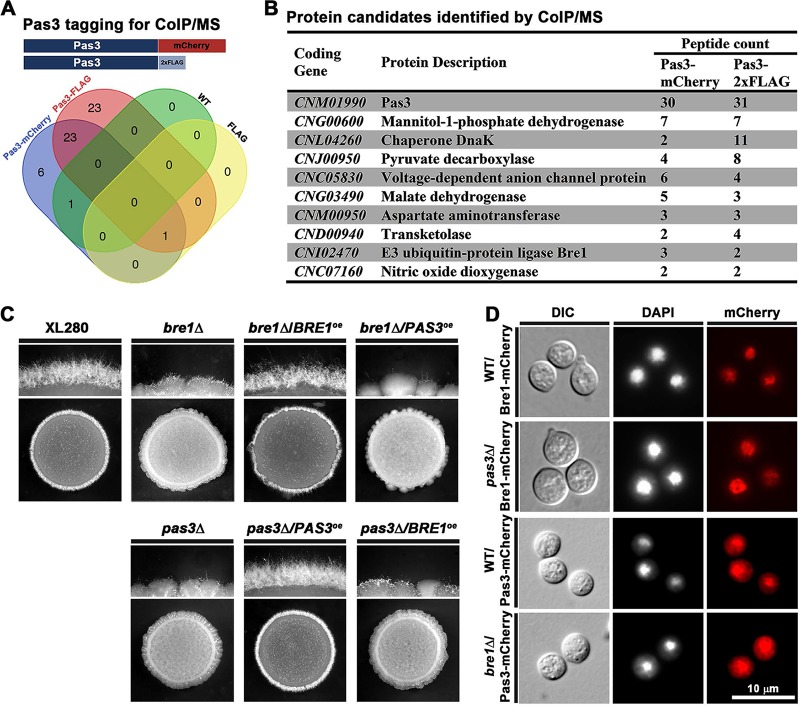
Pas3 interacts with Bre1 to regulate filamentation. (A) The diagram depicts the bait constructs of Pas3 tagged with either mCherry or 2×FLAG for Co-IP/MS. WT XL280 without any tag and a strain bearing just the 2×FLAG tag were included as negative controls. The Venn diagram shows all the overlapped protein candidates identified through Co-IP/MS by RFP and FLAG traps. (B) The list of protein candidates that are potential interacting partners of Pas3 sorted by the number of peptide counts identified by MS. In this refined list, the abundant proteins such as those in the translation machinery were excluded. (C) WT, *bre1*Δ, *bre1*Δ/*BRE1^oe^*, *bre1*Δ/*PAS3^oe^, pas3*Δ, *pas3*Δ/*PAS3^oe^*, and *pas3*Δ/*BRE1^oe^* strains were spotted onto V8 medium and cultured at 22°C in dark for 3 days. (D) Localization of Bre1, please correct the definition.or Pas3 in the absence of *PAS3* or *BRE1*. DAPI staining was used to visualize the nucleus. DIC, differential inference contrast.

Given that Pas3 has no predicted domains other than the PAS domain, we hypothesized that Pas3 interacts with its partners to regulate cryptococcal filamentation. To identify proteins interacting with Pas3, we performed coimmunoprecipitation (Co-IP) experiments with a Pas3-mCherry strain and a Pas3-FLAG strain using RFP trap or FLAG trap (Fig. 5A). A strain with an empty FLAG tag and the WT strain with no tag at all were included as negative controls. Proteins shared in the pulldowns from Pas3-mCherry and Pas3-FLAG, but absent in the negative controls, were considered to represent hits (Fig. 5A). We further prioritized the candidates, among those whose functions are known or predicted (Fig. 5B), based on potential nuclear localization and a role in transcription regulation. We eventually decided to focus on the Bre1 candidate.

Indeed, deletion of *BRE1* in the XL280 background dramatically reduced filamentation on V8 medium. Reintroduction of *BRE1* into the *bre1*Δ mutant complemented its filamentation defect (Fig. 5C), confirming the importance of Bre1 in regulating filamentation. As expected, mCherry-tagged Bre1 was localized in the nucleus (Fig. 5D). Given that Pas3 is enriched in the nucleus without an NLS, we decided to examine if the interaction with Bre1 affects the nuclear localization of Pas3. We introduced mCherry-labeled Pas3 into the *bre1*Δ mutant and found that Pas3 remained enriched in the nucleus, indicating that the interaction with Bre1 is not critical for its nuclear localization (Fig. 5D). Likewise, Bre1 was located in the nucleus with or without Pas3 (Fig. 5D). Therefore, Pas3 and Bre1 do not affect each other’s subcellular localization.

If the physical interaction between Pas3 and Bre1 were critical to control filamentation, we would expect that simple overexpression of one component would not be sufficient to compensate for the loss of the other. Indeed, neither overexpression of *PAS3* in the *bre1*Δ mutant nor overexpression of *BRE1* in the *pas3*Δ mutant could restore filamentation (Fig. 5C). This contrasts with the ability of *ZNF2* overexpression to confer filamentation in the *pas3*Δ mutant (Fig. 3A). Taking the data together, Pas3 interacts with Bre1 in regulating cryptococcal filamentation.

### Bre1 is required for H2Bub1 and H3K4me2, and Bre1 and Pas3 share similar regulons involved in regulating cryptococcal filamentation.

**FIG 6:**
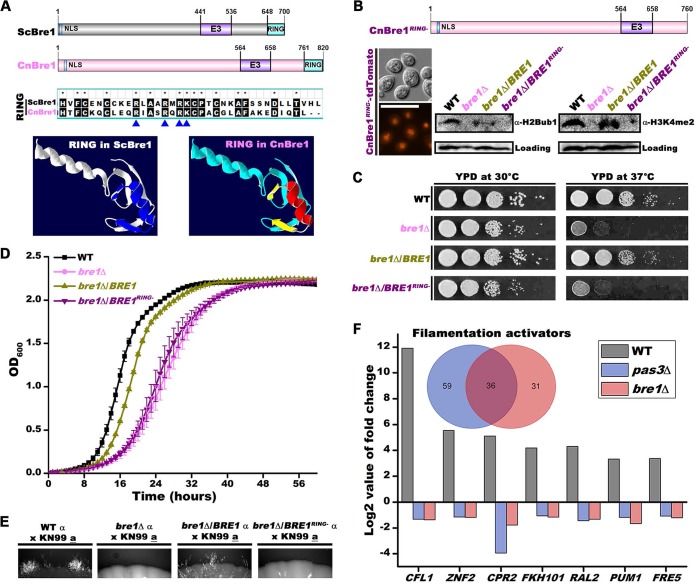
Bre1-mediated H2Bub1 and H3K4me2 are essential for its function in C. neoformans, and Bre1 regulon largely overlaps Pas3. (A) S. cerevisiae and C. neoformans Bre1 homologs are conserved in terms of domain structure. The structure of Bre1’s RING domain (pdb4r7e) in S. cerevisiae was used as a template for homology modeling via SWISS-MODEL. (B) The RING domain of Bre1 is essential for its function in mediating H2Bub1 and H3K4me2 in C. neoformans. Bre1*^RING^*^−^ was tagged with tdTomato and introduced into the *bre1*Δ mutant. The samples for Western blotting were collected from overnight cultures in liquid YPD medium at 30°C with shaking at 220 rpm. (C) Spotting assay on YPD agar medium showing the impact of *BRE1* deletion on cell growth at 30°C and 37°C in C. neoformans. (D) Growth curve of the cryptococcal strains in liquid YPD at 37°C. (E) The RING domain of Bre1 regulates unilateral bisexual filamentation in the serotype A strain background. The wild-type strain and the *bre1*Δ, *bre1*Δ/*BRE1*, and *bre1*Δ/*BRE1^RING^*^−^ mutant strains were crossed with the KN99 **a** strain on a V8 (pH 5) plate in dark at 22°C, and hyphal formation was determined after 10 days. (F) A Venn diagram of filamentation-associated genes that are activated by Pas3 and Bre1. The bar graph shows the differential expression levels of genes with known functions that are commonly regulated by Pas3 and Bre1.

The Bre1 homolog in S. cerevisiae is an E3 ubiquitin ligase. Cryptococcal Bre1 and *Saccharomyces* Bre1 share similar domain arrangements, and comparisons of the RING domains showed that they are highly conserved in sequence (Fig. 6A). Homology modeling based on the RING domain structure of *Saccharomyces* Bre1 (pdb4r7e) showed identical RING domain structures in the two homologs (Fig. 6A). In S. cerevisiae, Bre1 regulates gene transcription by monoubiquitinating histone H2B (H2Bub1), which subsequently affects histone H3 lysine 4 dimethylation (H3K4me2) (42). We found that the deletion of *BRE1* in C. neoformans abolished monoubiquitination in H2B on the basis of Western blot analyses probed with an H2Bub1 antibody (Fig. 6B). Furthermore, H3K4me2 was also abolished in the *bre1*Δ mutant on the basis of Western blot analyses probed with an H3K4me2 antibody (Fig. 6B). The results indicate the critical role of *Cryptococcus* Bre1 in mediating H2Bub1 and H3K3me2, as previously demonstrated for *Saccharomyces* Bre1. Thus, despite the divergence of these two fungal species (basidiomycetes versus ascomycetes), Bre1 appears to be conserved in structure and function. In S. cerevisiae, deletion of *BRE1* causes growth defect (43) (Fig. S3A). Consistently, we observed a similar defect in growth of the cryptococcal *bre1*Δ mutant (Fig. 6C and D). Overexpression of *BRE1* in mutant *bre1*Δ partially restored the levels of H2Bub1 and H3K4me2 and the cryptococcal growth rate (Fig. 6B and C and D). Bre1 with an in-frame deletion of the RING domain (*BRE1^RING^*^−^) failed to restore either the levels of H2Bub1 and H3K4me2 or the growth defect in mutant *bre1*Δ (Fig. 6B and C and D). This again corroborates the previous report demonstrating the critical contribution of the RING domain to Bre1’s function in S. cerevisiae (43). Taking the data together, these results establish the functional conservation of Bre1 in H2Bub1 and H3K4me2 and of cell growth in these two highly divergent fungal species.

10.1128/mBio.02135-18.3 FIG S3 Bre1 regulates cell growth in C. neoformans. (A) Serially diluted cells (starting from OD_600_ = 3) were spotted onto YPD agar medium and grown at the indicated temperature for 2 days. (B and C) Diluted cells (starting from OD_600_ = 0.03) were inoculated into 24-well plates, and cell growth rates were monitored via Cytation 5. Download FIG S3, PDF file, 0.2 MB. Copyright © 2018 Zhao et al. 2018 Zhao et al. This content is distributed under the terms of the Creative Commons Attribution 4.0 International license.

Deletion of *BRE1* in the serotype D strain XL280 background dramatically reduced self-filamentation (Fig. 5C). To investigate the impact of deleting the RING domain of Bre1 on cryptococcal filamentation, we tested unilateral mating hyphal formation in serotype A strain background. Consistent with what we had observed in the serotype D background, deletion of *BRE1* severely reduced unilateral filamentation relative to wild-type control results and overexpression of *BRE1* partially restored the unilateral filamentation (Fig. 6E). Interestingly, Bre1 with an in-frame deletion of the RING domain (*BRE1*^RING−^) failed to restore the filamentation defect (Fig. 6E). These results strongly suggest that Bre1-mediated histone modifications regulate filamentation in C. neoformans.

Given that Pas3 works with Bre1 to control filamentation, we reasoned that Bre1 and Pas3 may share common filamentation-associated regulons, especially for the filamentation activators. Comparing the transcriptome of mutant *bre1*Δ with that of the WT strain cultured on V8 medium, we identified 175 differentially expressed genes (79 upregulated and 96 downregulated). Combined with the transcriptome of WT V8 versus WT YPD, we identified 67 of 96 downregulated genes in the mutant as potential Bre1-regulated activators of filamentation and 27 of 75 upregulated genes as suppressors of filamentation (Table S5). More than half of the potential filamentation activators (36 of 67) are shared by both Bre1 and Pas3 (Fig. 6F). The degrees of reduction in the transcript levels of these shared genes, including *ZNF2* and its known downstream targets such as *CFL1*, were strikingly similar in comparisons between the *bre1*Δ and *pas3*Δ mutants (Fig. 6F). These results are consistent with the idea that Bre1 and Pas3 function together in regulating filamentation-associated genes at the transcript level.

## DISCUSSION

C. neoformans is a ubiquitous environmental microbe and an opportunistic pathogen of a wide range of hosts. Its success relies on its ability to sense and respond to multiple and changing environmental cues. PAS proteins are known to function in signal sensing, transduction, and regulation. Three PAS proteins in C. neoformans, Bwc1, Bwc2, and Tco2, regulate light sensing and other physiological changes (15, 19). Here, we systematically characterized all nine PAS proteins encoded in the C. neoformans genome for their role in regulating cryptococcal virulence traits and filamentation. We discovered that deletion of *PAS3* impairs melanization but enhances capsule production. The *pas3*Δ mutants in both the serotype A and serotype D backgrounds were fully virulent in a murine model of cryptococcosis. This likely reflects the opposing effects of Pas3 on melanization and capsule production and the complex interactions between different traits that could shape the outcome of the infection.

Pas3 regulates both cryptococcal cell fusion and filamentation, but it is not critical for sporulation. Given that it is a small protein lacking NLS or a DNA binding domain, we were surprised that Pas3 is enriched in the nucleus and that its nuclear localization is independent of the (nutrient-rich and mating-, capsule-, and melanin-inducing) culture conditions that we tested. We postulate that the PAS domain may play a critical role in its subcellular distribution and function. Indeed, when the integrity of the PAS domain is compromised, due either to mutations of specific residues (G72A and Y73A) or to an in-frame deletion of the core sequence within the domain, nuclear enrichment of Pas3 is impaired or abolished. These core residues are known to contribute to protein-protein interactions among PAS proteins in other organisms (13, 14, 41, 44). However, we did not detect any interaction between Pas3 and four other potentially nucleus-localized PAS proteins on the basis of results from the yeast two-hybrid (Y2H) assay (see Fig. S4 in the supplemental material). This suggests that Pas3 may interact with non-Pas partners.

10.1128/mBio.02135-18.4 FIG S4 Pas3 does not interact with other PAS proteins that are potentially localized in the nucleus. The full-length cDNAs of *BWC1* and *PAS3* in strain H99 were cloned into bait vector pGBKT7 and fused with the BD domain. The cDNAs of *BWC2*, *PAS1*, and *PAS2* were cloned into prey vector pGADT7 and fused with the AD domain. Both bait constructs and prey constructs were cotransformed into yeast strain PJ69-4A. Transformants growing on SD medium lacking histidine and adenine were considered to be representative of positive interactions. The interaction between Bwc1 and Bwc2 served as the positive control. Download FIG S4, PDF file, 0.2 MB. Copyright © 2018 Zhao et al. 2018 Zhao et al. This content is distributed under the terms of the Creative Commons Attribution 4.0 International license.

We hypothesize that Pas3’s interacting partner(s) must aid its recruitment to the nucleus and its regulation of filamentation. Our current evidence suggests that different partners likely carry out these tasks. One of the partners is Bre1, an E3 ligase with a conserved role in monoubiquitination of histone H2B (H2Bub1). H2Bub1 is required for histone H3K4me2 (45–50), which is tightly associated with promoters of actively transcribed genes (51). Accordingly, H2Bub1 is associated with actively transcribed genes in fungi, plants, and humans. Indeed, we found that Bre1 in *Cryptococcus* has the same functions as Bre1 in S. cerevisiae (43). Strikingly, Bre1 and Pas3 share common regulons that are associated with filamentation, and both gene deletion mutants are severely defective in filamentation. One of many potential models that could explain our current findings is that Pas3 guides Bre1 to filamentation-associated loci and promotes the transcription of *ZNF2* and its prominent downstream targets under certain conditions. Although the mode of action of Pas3-Bre1 is yet to be established, the findings presented in this study clearly demonstrate an interesting layer of regulation on cryptococcal morphogenesis through histone modification directed by signaling factors.

## MATERIALS AND METHODS

### Strains and growth conditions.

The strains and plasmids used in this study are listed in Table S1 in the supplemental material. Yeast cells were grown on YPD medium unless specified otherwise. Mating and filamentation assays were conducted on V8 agar medium in the dark at 22°C as previously described (11). Transformants obtained by biolistic transformation (52) or TRACE (53) were selected on YPD with 100 μg/ml of nourseothricin (NAT), 100 μg/ml of neomycin (NEO), or 200 μg/ml of hygromycin (HYG).

10.1128/mBio.02135-18.5 TABLE S1 Strains, plasmids, and primers used in this study. Download Table S1, DOCX file, 0.03 MB. Copyright © 2018 Zhao et al. 2018 Zhao et al. This content is distributed under the terms of the Creative Commons Attribution 4.0 International license.

10.1128/mBio.02135-18.6 TABLE S2 DE genes of WT V8 versus WT YPD. Download Table S2, XLSX file, 0.1 MB. Copyright © 2018 Zhao et al. 2018 Zhao et al. This content is distributed under the terms of the Creative Commons Attribution 4.0 International license.

10.1128/mBio.02135-18.7 TABLE S3 Znf2-regulated filamentation regulon. Download Table S3, XLSX file, 0.02 MB. Copyright © 2018 Zhao et al. 2018 Zhao et al. This content is distributed under the terms of the Creative Commons Attribution 4.0 International license.

10.1128/mBio.02135-18.8 TABLE S4 Pas3-regulated filamentation regulon. Download Table S4, XLSX file, 0.02 MB. Copyright © 2018 Zhao et al. 2018 Zhao et al. This content is distributed under the terms of the Creative Commons Attribution 4.0 International license.

10.1128/mBio.02135-18.9 TABLE S5 Bre1-regulated filamentation regulon. Download Table S5, XLSX file, 0.02 MB. Copyright © 2018 Zhao et al. 2018 Zhao et al. This content is distributed under the terms of the Creative Commons Attribution 4.0 International license.

### Gene manipulation.

To delete the *PAS3* open reading frame (ORF), the deletion construct, consisting of approximately 1-kb flanking sequences and the split dominant marker NAT, was introduced into the indicated recipient strain by biolistic transformation as described previously (52). The transformants generated were screened by two rounds of diagnostic PCR. The first round of PCR was performed to detect integration of the construct into the correct locus (primer pair Linlab2271 and P-*actin* reverse for the H99 background and primer pair Linlab2837 and P-*actin* reverse for the XL280 background). The second round of PCR was performed to confirm the loss of the *PAS3* ORF (primer pair Linlab2902 and Linlab2903 for the H99 background and primer pair Linlab2904 and Linlab2905 for the XL280 background). Mutants that passed the PCR diagnostic tests were then selected for the genetic linkage assay using meiotic progeny dissected from genetic crosses as described previously (54). The deletion mutant in the *MAT***a** background was obtained by crossing the *MAT*α *pas3*Δ strains with the corresponding congenic WT **a** strain, as described previously (37).

For complementation, the *PAS3* ORF and 1.5 kb of upstream region were amplified by PCR, digested, and cloned into pXL1 without the *GPD1* promoter to generate pYZ1 (12). For gene overexpression, the constructs were created by amplifying the entire ORF by PCR and cloning them into pXL1 behind the *GPD1* promoter.

To tag a protein, the ORF was amplified by PCR and cloned into the pXL1 backbone vector with a mCherry tag or a 2×FLAG tag driven by the *GPD1* promoter. The tagged constructs were introduced into the recipient strains either by biolistic transformation (52) or by TRACE (53). All primers and plasmids used in this study are listed in Table S1.

To introduce single nucleotide mutations in the *PAS3* gene, a QuikChange II XL site-directed mutagenesis kit (from Agilent Technologies) was used following the manufacturer’s instructions. In brief, the *PAS3* ORF was amplified by PCR and cloned into a pGEM-T easy vector (Promega). The entire plasmid with the insertion was then amplified using specific primers to change the codons of conserved residues. Primers for introducing the mutations are included in Table S1. The modified insertions were then amplified from the plasmid using PCR with high-fidelity Phusion master mix (Life Technology) and cloned into the pXL1 backbone (12) with either a mCherry or 2×FLAG tag. The mutated and tagged *PAS3* alleles were then introduced into the *pas3*Δ mutant or the WT strain via biolistic transformation. Stable transformants were selected after stability testing with five passages on nonselective medium and further analyzed by diagnostic PCR to confirm the replacement. In-frame deletions of the core sequence of the PAS domain in Pas3 and the RING domain in Bre1 were achieved via fusion PCR. All the primers used in this study were listed in Table S1.

### Microscopic examination.

The mCherry-tagged strains were cultured overnight in YPD and observed under a Zeiss Imager M2 microscope. Images were acquired with an AxioCam MRm camera and processed with Zen 11 software (Carl Zeiss Microscopy). Nuclei were visualized using 10 μg/ml of DAPI (4′,6-diamidino-2-phenylindole) after fixation as described previously (55).

### Bioinformatics and phylogenetic analysis.

To identify the PAS domain proteins in the selected fungi, “PAS” was used as the keyword and the PAS domain protein sequences from C. neoformans H99 and JEC21 were used as the query sequences for blastp searches against the selected fungal genomes on FungiDB (56). Phylogenetic analyses for these PAS proteins were conducted with their whole-protein sequences by using the neighbor-joining method in MEGA7 (57).

### *In vitro* phenotypic assays.

Strains to be tested were grown overnight in liquid YPD at 30°C with shaking. The cells were washed, adjusted to the same cell density (optical density at 600 nm [OD_600_] = 3.0), and serially diluted. To analyze melanization, the cell suspensions with the dilutions were spotted onto agar media containing l-dihydroxyphenylalanine (l-DOPA) and incubated at 37°C in the dark. To observe capsule production, cells were spotted onto low-iron medium (LIM) (58) and grown at 37°C at 5% CO_2_. The capsule was visualized by India ink exclusion and examined under a light microscope. To test the ability of the strains to grow at high temperatures and their sensitivity to UV radiation, equal numbers of cells were spotted onto yeast nitrogen base (YNB) agar medium and grown at 37°C with or without exposure to 300 J/m^2^ of UV (3s). Cells were then incubated at 30°C for additional 2 days. To test the tolerance of fungal cells to osmotic stresses and other stressors, cells were grown at 22°C for 2 days on YPD medium with or without supplementation with H_2_O_2_ (21 mM), NaCl (1.5 M), fludioxonil (100 μg/ml), or Congo red (200 μg/ml). To test the growth rate, cells were inoculated into 24-well plates containing 1 ml of YPD liquid medium/well at a starting cell density of OD_600_ = 0.03. The plates were incubated at 30°C or 37°C for 48 or 60 h with orbital shaking. The OD_600_ of each well was measured every hour with a Cytation 5 (BioTek) multimode plate reader.

### Murine macrophage phagocytosis assay.

A murine macrophage phagocytosis assay was conducted using a procedure that we previously described (8, 59). Mouse macrophage cell line J774A.1 (ATCC TIB-67TM) was acquired from the American Type Culture Collection, along with ATCC-formulated Dulbecco's modified Eagle's medium (DMEM) (catalog no. 30-2002). Fetal bovine serum (FBS) was added into DMEM to reach a final concentration of 10% immediately prior to inoculation. Freshly grown J774A.1 cells (300 μl) were seeded into 24-well microtiter plates, with 2.5 × 10^5^ cells per well. The macrophage cells were cultured at 37°C with 5% CO_2_ overnight. Old culture medium was replaced by fresh DMEM with 10% FBS. *Cryptococcus* cells (2.5 × 10^6^) were inoculated into each well with calcofluor white (20 μg/ml) for the phagocytosis assay. After 30 s of shaking and mixing, the cocultures were incubated at 37°C with 5% CO_2_ for 2 h. The cocultures were then washed three times with warmed phosphate-buffered saline (PBS; 500 μl/well/wash) to remove nonadherent cells prior to fixation with 300 μl 10% formaldehyde–PBS. The cells were examined under an inverted microscope (Eclipse Ti; Nikon), and the images were captured using the NIS elements AR 3.0 software.

### Cryptococcal fluconazole protection assay.

The fluconazole protection assay was used to measure intracellular replication of *Cryptococcus* cells in mouse macrophage cell line J774A.1 (ATCC TIB-67TM) as we previously described (60). We infected macrophage cells with *Cryptococcus* cells (2.5 × 10^6^) for 2 h, performed extensive washes (6 to 8 times), and then added fresh medium containing fluconazole (18 µg/ml). The cells were then incubated for another 12 h. The medium was then removed, and the macrophage cells were lysed by the addition of 0.5% Tween 20 in sterile water (100 µl) and incubated at 37°C for 10 min. The resultant whole-cell lysates (which contained the intracellular *Cryptococcus* population) were plated onto solid YPD medium after serial dilution. CFU counts were performed after 48 h of incubation at 30°C.

### Murine cryptococcosis and lung fungal burden analysis.

Female A/J mice (8 to 10 weeks old) were purchased from the Jackson Laboratory (Bar Harbor, ME). Cryptococcal strains were cultured in YPD liquid medium overnight at 30°C with shaking at 220 rpm. The fungal cells were washed with sterile saline solution three times and adjusted to 2 × 10^5^ cell/ml for serotype A strains and 2 × 10^7^ cells/ml for serotype D strains. Mice were sedated with ketamine and xylazine via intraperitoneal injection and then inoculated intranasally with 50 μl fungal cell suspension (1 × 10^4^ cells/animal for serotype A strains and 1 × 10^6^ cells/animal for serotype D strains). After infection, animals were monitored daily for disease progression, including weight loss, gait changes, labored breaths, or fur ruffling. The animal experiments were carried out in strict accordance with the recommendations in the Guide for the Care and Use of Laboratory Animals of the National Institutes of Health and IACUC regulation. The mice were euthanized after 12 days postinfection with serotype A strains and 17 days postinfection with serotype D strains (60). Lungs were dissected and homogenized in 2 ml cold PBS buffer. The tissue suspensions were serially diluted, plated onto YNB agar medium, and incubated at 30°C for 2 days for CFU counting.

### Cell-cell fusion assay.

YSB44 (H99α *can1*::*NAT*) and YSB133 (KN99a *can1*::*NEO*) were used as genetically marked wild-type strains to study the fusion competency of *mat2*Δ (Linlab2922), *znf2*Δ (Linlab3066), and *pas3*Δ (YZ3) mutants. Strains for each fusion pair were grown overnight in YPD liquid medium at 30°C. Cells were washed twice with distilled water and diluted to a final density of OD_600_ = 3. After that, 50 μl of equal-volume mixed cells were spotted onto V8 (pH 5) medium and incubated for 48 h in the dark at 22°C. The cells were then collected, washed with double-distilled water (ddH_2_O), and plated onto YPD medium or YPD medium supplemented with both NAT and G418. Colonies that appeared on medium containing both drugs after incubation at 22°C for 5 days were considered fusion products. The cell-cell fusion frequency between control strains YSB44 and YSB133 was set as 100% for normalization.

### RNA purification and qPCR.

RNA extraction and real-time quantitative PCR (qPCR) were carried out using procedures that we had previously described (12). Briefly, total RNA was extracted using a PureLink RNA minikit (Life Technologies). DNase (Ambion)-treated RNA samples were analyzed on a denaturing formaldehyde agarose gel for assessing purity, quality, and concentration. A SuperScript III cDNA synthesis kit (Life Technology) was used for the first-strand cDNA synthesis following the manufacturer’s protocols. Constitutively expressed housekeeping gene *TEF1* was used as an endogenous control for the normalization of expression of the other genes studied.

### RNA-sequencing (RNA-seq) and data analysis.

Direct poly(A) RNA sequencing was performed by the Texas A&M AgriLife Genomics and Bioinformatics Service. Preliminary quality analysis of the raw FASTQ files was conducted using FastQC (61), and trimming of low-quality bases was performed with a custom perl script. Tophat2 (62) was used to map the processed reads to the reference genome. Transcriptome data were determined from the genome annotation. The program htseq-count (63) and DESeq2 (64) were used to count reads and identify differentially expressed genes.

### Yeast two-hybrid assay.

Yeast two-hybrid assays were performed as previously described (65). The full-length cDNAs of *BWC1* and *PAS3* in strain H99 were cloned into bait vector pGBKT7 and fused with the BD domain. The cDNAs of *BWC2*, *PAS1,* and *PAS2* were cloned into prey vector pGADT7 and fused with the AD domain. All inserted cDNA sequences were confirmed by sequencing. Bait constructs and prey constructs were cotransformed into yeast strain PJ69-4A. Transformants growing on Sabouraud dextrose (SD) medium lacking histidine and adenine were considered to be representative of positive interactions.

### Protein extraction, Western blotting, and Co-IP/MS.

Proteins were extracted from the WT strain and from strains with Pas3-2×FLAG, Pas3-mCherry, Pas3^G72A^-2×FLAG, Pas3 ^G72A^-mCherry, Pas3^Y73A^-2×FLAG, Pas3 ^Y73A^-mCherry, and Pas3-2×FLAG/Bre1-mCherry following a previously described method (66). Aliquots of proteins were separated on 12% SDS-PAGE gels and then transferred to a polyvinylidene difluoride (PVDF) membrane for Western blot analysis using anti-FLAG (Sigma) or anti-RFP (Roche) monoclonal antibody. Protein aliquots from FLAG-tagged or mCherry-tagged strains were processed for Co-IP using FLAG trap (Sigma) or RFP trap (ChromoTek) and following the manufacturer's protocol. For Co-IP/mass spectroscopy (Co-IP/MS) assays, Co-IP samples were sent to the University of Texas (UT) proteome facility center for mass spectroscopy (MS). We first excluded abundant proteins that often show up in mass spectrometry assays, such as factors of the translation machinery and of other primary metabolic pathways. We focused on the candidates that have potential nuclear localization.

### Data availability.

The RNA-seq data have been deposited in the SRA database of NCBI (https://www.ncbi.nlm.nih.gov/sra). The SRA identifier (ID) of *ZNF2*-related RNA-seq data is SRP056166. The *PAS3-* and *BRE1*-related RNA-seq data can be found under BioProject PRJNA471863.
