# Supplementary figures and images for: PAS Domain Protein Pas3 Interacts with the Chromatin Modifier Bre1 in Regulating Cryptococcal Morphogenesis
Source: mBio. 2018 Nov 13;9(6):e02135-18. doi: 10.1128/mBio.02135-18 (PMC6234864; doi:10.1128/mBio.02135-18)

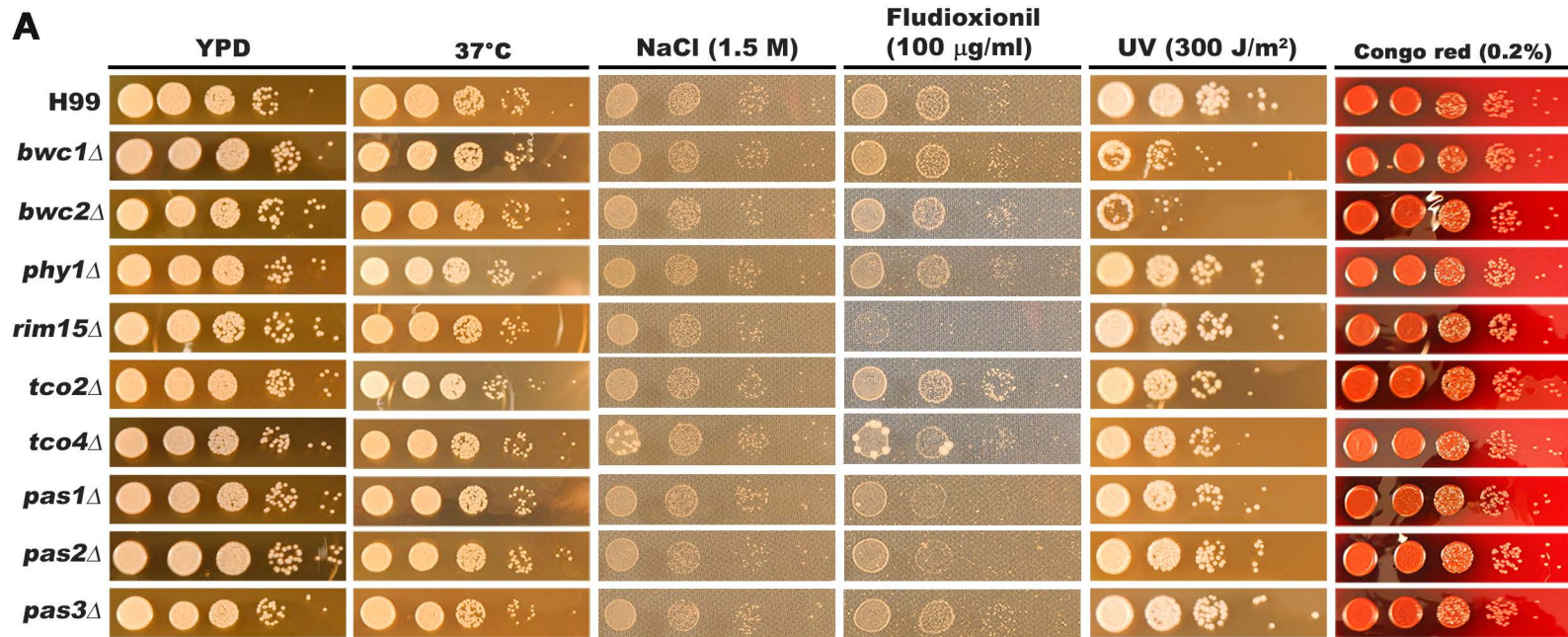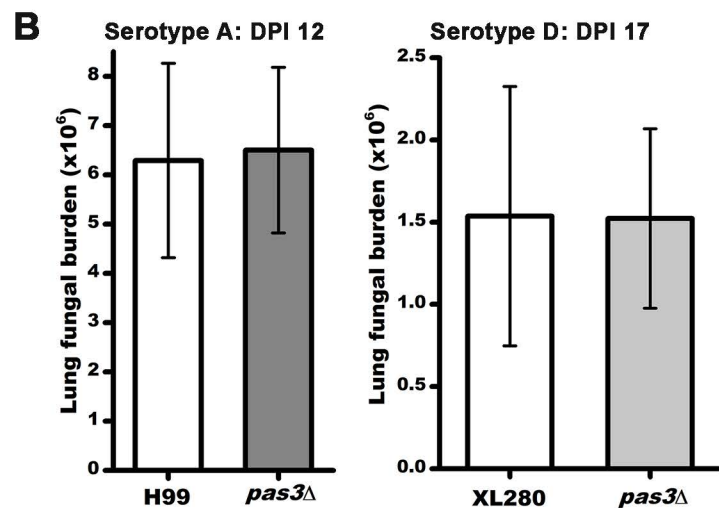

Supplement: FIG S1 [file mbo005184156sf1.pdf]

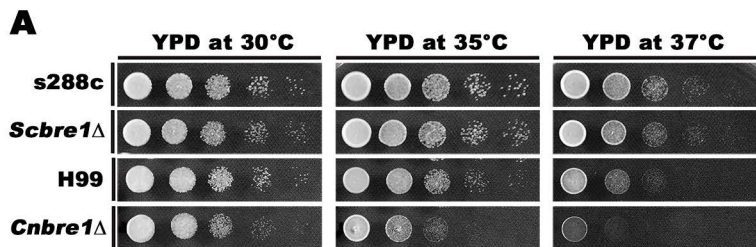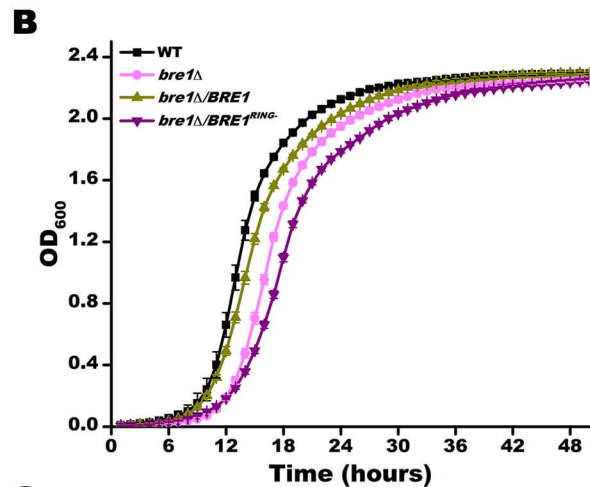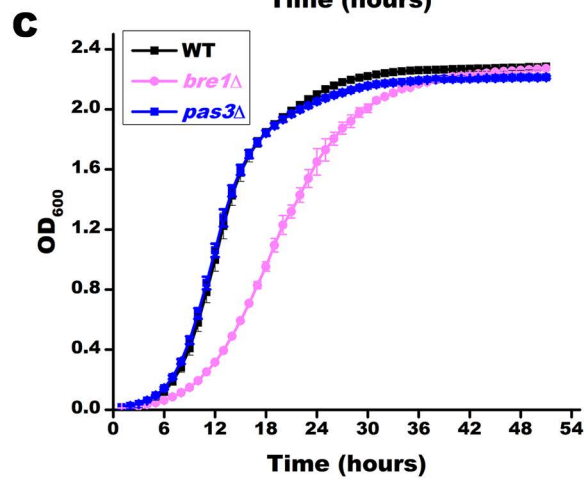

Supplement: FIG S3 [file mbo005184156sf3.pdf]

**BD Bwc1**  
**AD Bwc2**

**BD Bwc2**  
**AD Bwc1**

**BD Pas3**  
**AD Pas1**

**BD Pas3**  
**AD Pas2**

**BD Pas3**  
**AD Bwc2**

**-his**  
**-ade**

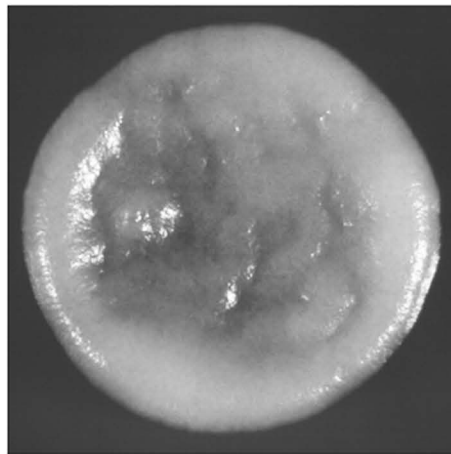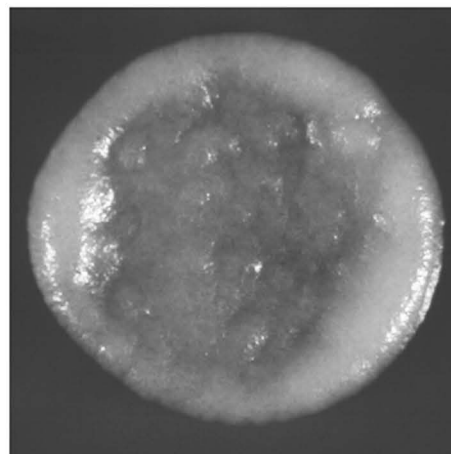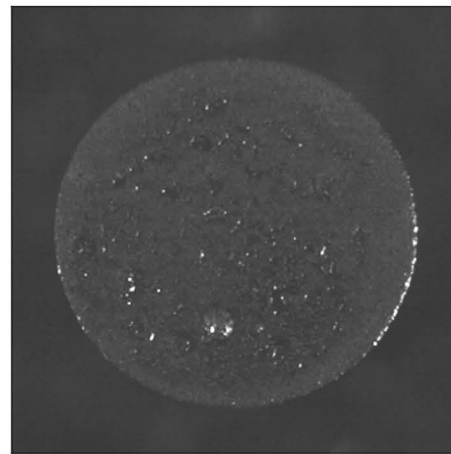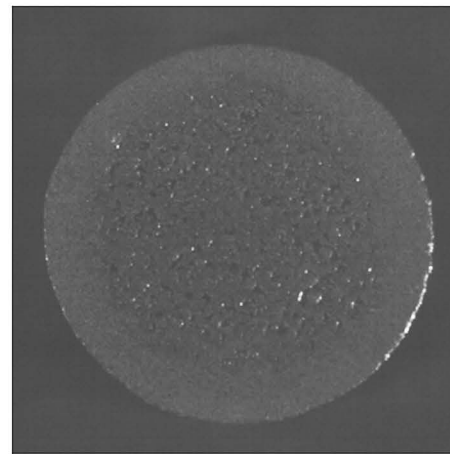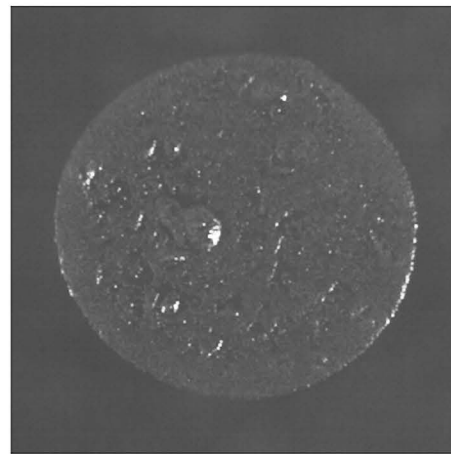

Supplement: FIG S4 [file mbo005184156sf4.pdf]
