# Supplementary material for: PAS Domain Protein Pas3 Interacts with the Chromatin Modifier Bre1 in Regulating Cryptococcal Morphogenesis
Source: mBio. 2018 Nov 13;9(6):e02135-18. doi: 10.1128/mBio.02135-18 (PMC6234864; doi:10.1128/mBio.02135-18)
Supplement: FIG S2 [file mbo005184156sf2.pdf]

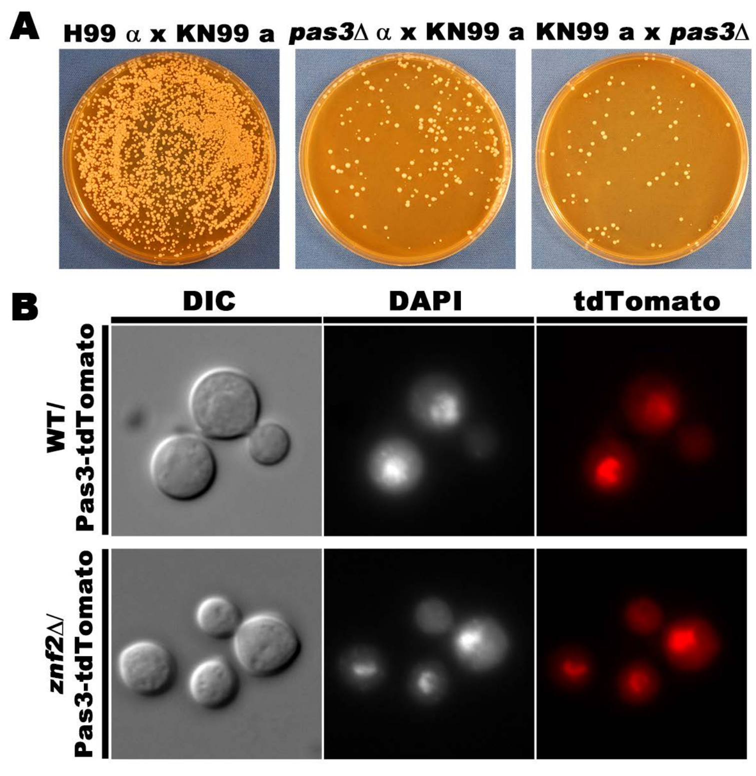
A
H99 α x KN99 a
pas3Δ α x KN99 a
KN99 a x pas3Δ
B
DIC
DAPI
tdTomato
WT/
Pas3-tdTomato
znf2Δ/
Pas3-tdTomato
