# Supplementary material for: PAS Domain Protein Pas3 Interacts with the Chromatin Modifier Bre1 in Regulating Cryptococcal Morphogenesis
Source: mBio. 2018 Nov 13;9(6):e02135-18. doi: 10.1128/mBio.02135-18 (PMC6234864; doi:10.1128/mBio.02135-18)
Supplement: TABLE S1 [file mbo005184156st1.docx]

**Supplementary Table S1. Strains, plasmids and primers used in this study.**

| **Strain name** | **Genotype** | **Background** | **Sources and comments** |
| --- | --- | --- | --- |
| *C. neoformans* |  |  |  |
| H99 | WT | H99α | (1) |
| KN99 | WT | KN99**a** | (1) |
| YZ268 | *bwc1*::*NAT* | H99α | Madhani lab |
| YZ269 | *bwc2*::*NAT* | H99α | Madhani lab |
| YZ270 | *phy1*::*NAT* | H99α | Madhani lab |
| YZ271 | *rim15*::*NAT* | H99α | Madhani lab |
| YZ272 | *tco2*::*NAT* | H99α | Madhani lab |
| YZ273 | *tco4*::*NAT* | H99α | Madhani lab |
| YZ274 | *pas1*::*NAT* | H99α | Madhani lab |
| YZ275 | *pas2*::*NAT* | H99α | Madhani lab |
| YZ3 | *pas3*::*NAT* | H99α | This study |
| YSB44 | *can1*::*NAT* | H99α | (2) |
| YSB133 | *can1*::*NEO* | KN99**a** | (2) |
| Linlab2922 | *mat2*::*NAT* | H99α | (3) |
| Linlab3066 | *znf2*::*NAT* | H99α | (3) |
| YZ634 | *PGPD1-BRE1-mCherry-NEO* | H99α | This study |
| XL280α | WT | XL280α | (4) |
| XL280**a** | WT | XL280**a** | (5) |
| YZ36 | *pas3*::*NAT* | XL280α | This study |
| YZ38 | *pas3*::*NAT* | XL280**a** | This study |
| YZ47 | *pas3*::*NAT*, P*_PAS3_*-*PAS3*-2x*FLAG*-*HYG* | XL280α | This study |
| YZ345 | *pas3*::*NAT*, P*_GPD1_*-*PAS3*-*mCherry*-*NEO* | XL280α | This study |
| YZ122 | P*_TEF1_*-*PAS3*-*tdTomato*-*NEO* | XL280α | This study |
| YZ346 | *pas3*::*NAT*, P*_GPD1_*-*PAS3^G72A^*-*mCherry*-*NEO* | XL280α | This study |
| YZ347 | *pas3*::*NAT*, P*_GPD1_*-*PAS3^Y73A^*-*mCherry*-*NEO* | XL280α | This study |
| YZ147 | *pas3*::*NAT*, P*_CTR4_*-*mCherry*-*ZNF2*-*NEO* | XL280α | This study |
| XL574 | *znf2*::*NAT* | XL280α | (3) |
| XL904 | *znf2*::*NAT*, P*_GPD1_*-*ZNF2*-*NEO* | XL280α | (3) |
| YZ195 | *znf2*::*NAT*, P*_TEF1_*-*PAS3*-*tdTomato*-*NEO* | XL280α | This study |
| XX17 | *znf2*::*NAT*, P*_CTR4_*-*mCherry*-*ZNF2*-*NEO* | XL280α | (6) |
| YZ225 | *bre1*::NAT | XL280α | This study |
| YZ850 | *bre1*::NAT, P*_TEF1_*-*BRE1*-*2xFALG*-*HYG* | XL280α | This study |
| YZ839 | *bre1*::*NAT*, P*_GPD1_*-*PAS3*-*mCherry*-*NEO* | XL280α | This study |
| *S. cerevisiae* |  |  |  |
| PJ69-4A | *MAT****a*** *trp1-901 leu2-3,112 ura3-52 his3-200 gal4Δ gal8Δ LYS2::GAL1-HIS3 GAL2-ADE2 met2::GAL7-lacZ* |  | (7) |
| **Plasmid** | **Genotype** | **Background** | **Sources and comments** |
| pXL1 | P*_GPD1_* *NEO* *KANA* | pXL1 | (8) |
| pYZ1 | P*_PAS3_*-*PAS3*-2x*FLAG*-*HYG* | pXL1 | This study |
| pYZ2 | P*_GPD1_*-*PAS3*-mCherry-*NEO* | pXL1 | This study |
| pYZ3 | P*_TEF1_*-*PAS3*-*tdTomato*-*NEO* | pXL1 | This study |
| pYZ4 | P*_GPD1_*-*PAS3^G72A^*-*mCherry*-*NEO* | pXL1 | This study |
| pYZ5 | P*_GPD1_*-*PAS3^Y73A^*-*mCherry*-*NEO* | pXL1 | This study |
| pYZ6 | P*_TEF1_*-*BRE1*-*2xFALG*-*HYG* | pXL1 | This study |
| pYZ7 | P*_GPD1_*-*BRE1*-mCherry-*NEO* | pXL1 | This study |

| **Primer name** | **Sequence (5’ to 3’)** | **Description** |
| --- | --- | --- |
| M13F | GTAAAACGACGGCCAGT | *NAT* and *NEO* cassette and TRACE constructs from pXL1 |
| M13R | CAGGAAACAGCTATGAC | *NAT* and *NEO* cassette and TRACE constructs from pXL1 |
| Linlab2271/YZ | ATT GAA CTT TGA AAG GGT AC | *PAS3* right flanking F in H99 |
| Linlab2272/YZ | CTG GCC GTC GTT TTA CGC AAG ATC AAC TGT TCA TCA TA | *PAS3* right flanking R in H99 |
| Linlab2273/YZ | GTC ATA GCT GTT TCC TGT GGG GCA TGG TAT CCA CT | *PAS3* left flanking F in H99 |
| Linlab2274/YZ | CGA TTT CTA TTC CGC GAC | *PAS3* left flanking R in H99 |
| Linlab2837/YZ | GAG GGA GGT GGT GAA GAG | *PAS3* right flanking F in XL280 |
| Linlab2838/YZ | CTG GCC GTC GTT TTA CCA TGC CTG GAC AAG AAT T | *PAS3* right flanking R in XL280 |
| Linlab2839/YZ | GTC ATA GCT GTT TCC TGA TCG CCA GAA CGA AGA CC | *PAS3* left flanking F in XL280 |
| Linlab2840/YZ | CAG CAG GCA AAG AGG TCA | *PAS3* left flanking R in XL280 |
| *P-actin R* | TGTGGATGCTGGCGGAGGATA | *Confirming PAS3 deletion* |
| Linlab2902/YZ | ATA GGC CGG CCA TGC CTA CTC AAA CTC TTG GC | *Confirming PAS3 deletion in H99 F* |
| Linlab2903/YZ | ATA GCG ATC GCT CCT GTC AGA TTT TCA ATC GC | *Confirming PAS3 deletion in H99 R* |
| Linlab2904/YZ | ATA GGC CGG CCA TGC CTG CTC AAA CTC TTG | *Confirming PAS3 deletion in XL280 F* |
| Linlab2905/YZ | ATA GCG ATC GCT CCT GTC AGA TTT TCA ATC G | *Confirming PAS3 deletion in XL280 R* |
| Linlab2686/YZ | ATA GCG GCC GCT TGC AGT GCT GTG GTT CA | *P-PAS3 F* |
| Linlab2687/YZ | ATC TTA ATT AAA TAG ACA GTA CCC TAC ACC C | *P-PAS3 R* |
| Linlab2703/YZ | ATA GGC CGG CCT ATA ATT TCA CGC TTC TCG | *O-PAS3 F* |
| Linlab4424/YZ | GCAAGATATTCTTGCATACACCCCATCCG | *PAS3G72A* F |
| Linlab4425/YZ | CGGATGGGGTGTATGCAAGAATATCTTGC | *PAS3G72A* R |
| Linlab4426/YZ | GCAAGATATTCTTGGAGCCACCCCATCCGATTTG | *PAS3Y73A* F |
| Linlab4427/YZ | CAAATCGGATGGGGTGGCTCCAAGAATATCTTGC | *PAS3Y73A* R |
| Linlab4010/YZ | CAGATTGTTGCTGCTGTTAGG | *BRE1* right flanking F in XL280 |
| Linlab4011/YZ | CTGGCCGTCGTTTTACGATTTCGCTGTTCAATTCGT | *BRE1* right flanking R in XL280 |
| Linlab4012/YZ | GTCATAGCTGTTTCCTGTGCTGTCGCCTCAATGTTCG | *BRE1* left flanking F in XL280 |
| Linlab4013/YZ | GCTCATTAAAGGTCCCAACG | *BRE1* left flanking R in XL280 |
| Linlab4044/YZ | AGCGTTTACTTGCTCGTTATG | *Confirming BRE1 deletion F* |
| Linlab4045/YZ | GAAGTCTGAAACCGGCGTAG | *Confirming BRE1 deletion F* |
| Linlab4064/YZ | ATAAGGCCGGCCATGAACGCAGACCTCAAAAG | *O-BRE1 F* |
| Linlab4063/YZ | ATAAGCGATCGCTTGCCAATACAGTGTTTGTA | *O-BRE1* R |
| Linlab2995/YZ | aattGAATTCATGCCTACTCAAACTCTTG | *PAS3Y2HF* |
| Linlab2996/YZ | aattGGATCCCTATCCTGTCAGATTTTCAA | *PAS3Y2HR* |
| Linlab2997/YZ | aattGAATTCATGGACCAGCCATCTGGCT | *PAS1Y2HF* |
| Linlab2998/YZ | aattGGATCCTTATAGCTCCCAAATCCGT | *PAS1Y2HR* |
| Linlab2999/YZ | aattGAATTCATGCAAGACCACCAATGG | *PAS2Y2HF and PCR confirmation* |
| Linlab3000/YZ | aattGGATCCTTATTGCGTCTCAGCAGG | *PAS2Y2HR and PCR confirmation* |
| Linlab3001/YZ | aattCCCGGGATGTCAACAAACCTCACTT | *BWC1Y2HF and PCR confirmation* |
| Linlab3002/YZ | aattGGATCCTCAAAATCCAATACCCCCA | *BWC1Y2HR and PCR confirmation* |
| Linlab3003/YZ | aattGAATTCATGTCCCTCCTCGCCGA | *BWC2Y2HF and PCR confirmation* |
| Linlab3004/YZ | aattGGATCCTCAAGACGGTTGCTTGT | *BWC2Y2HR and PCR confirmation* |
| Linlab3091/YZ | GTCATAGCTGTTTCCTGTCCGTGCTGAATCAGAGG | *Confirmation of PHY1 deletion from deletion set* |
| Linlab3092/YZ | GTTAGCCAGGCTCGACAT | *Confirmation of PHY1 deletion from deletion set* |
| Linlab3081/YZ | CACCATCCAATCCCACAG | *Confirmation of RIM15 deletion from deletion set* |
| Linlab3082/YZ | CTGGCCGTCGTTTTACGCATCTTGCGAGCTTCTG | *Confirmation of RIM15 deletion from deletion set* |
| Linlab3085/YZ | TACGGGAAACGGGAAGGA | *Confirmation of TCO4 deletion from deletion set* |
| Linlab3086/YZ | CTGGCCGTCGTTTTACACTCGGGCATAGGGCTCA | *Confirmation of TCO4 deletion from deletion set* |
| Linlab3095/YZ | GTCATAGCTGTTTCCTGGGGGATACATGATGCTGA | *Confirmation of TCO2 deletion from deletion set* |
| Linlab3096/YZ | CCTACCAAAACCAAAACC | *Confirmation of TCO2 deletion from deletion set* |
| Linlab2964/YZ | GTCATAGCTGTTTCCTGCTCG CAGCCACCATTTTC | *Confirmation of PAS1 deletion from deletion set* |
| Linlab2965/YZ | GCCGTTGTGGATTCTTTTAT | *Confirmation of PAS1 deletion from deletion set* |
| Linlab3554/YZ | TTTCGGCTTTAGTGGTGT | *Confirmation of PAS3 deletion from deletion set* |
| Linlab3555/YZ | CTGTCGATGGTGGTGATG | *Confirmation of PAS3 deletion from deletion set* |

**References**

1. **Nielsen K, Cox GM, Wang P, Toffaletti DL, Perfect JR, Heitman J.** 2003. Sexual cycle of Cryptococcus neoformans var. grubii and virulence of congenic a and alpha isolates. Infection and Immunity **71:**4831-4841.

2. **Bahn YS, Cox GM, Perfect JR, Heitman J.** 2005. Carbonic anhydrase and CO2 sensing during Cryptocloccus neoformans growth, differentiation, and virulence. Current Biology **15:**2013-2020.

3. **Lin X, Jackson JC, Feretzaki M, Xue C, Heitman J.** 2010. Transcription factors Mat2 and Znf2 operate cellular circuits orchestrating opposite- and same-sex mating in Cryptococcus neoformans. PLoS Genet **6:**e1000953.

4. **Lin XR, Hull CM, Heitman J.** 2005. Sexual reproduction between partners of the same mating type in Cryptococcus neoformans. Nature **434:**1017-1021.

5. **Zhai B, Zhu PK, Foyle D, Upadhyay S, Idnurm A, Lin XR.** 2013. Congenic Strains of the Filamentous Form of Cryptococcus neoformans for Studies of Fungal Morphogenesis and Virulence. Infection and Immunity **81:**2626-2637.

6. **Xu XP, Li JF, Zhao YB, Kirkman E, So YS, Bahn YS, Lin XR.** 2017. Glucosamine stimulates pheromone-independent dimorphic transition in Cryptococcus neoformans by promoting Crz1 nuclear translocation. Plos Genetics **13**.

7. **James P, Halladay J, Craig EA.** 1996. Genomic libraries and a host strain designed for highly efficient two-hybrid selection in yeast. Genetics **144:**1425-1436.

8. **Wang L, Zhai B, Lin X.** 2012. The link between morphotype transition and virulence in Cryptococcus neoformans. PLoS Pathog **8:**e1002765.
